# CD8^+^ TILs in necrotic tumors after neoadjuvant immunochemotherapy predict outcomes in non-small-cell lung cancer patients

**DOI:** 10.1038/s41392-025-02435-0

**Published:** 2025-10-09

**Authors:** Haifeng Lin, Yi Han, Lei Guo, Caigang Liu, Hefei Li, Jie Li, Chong Wang, Lijuan Zhou, Xiangna Zhang, Lisha Sun, Ying Yi Zhang, Xiaojing Chu, Jianquan Shi, Xiaoqing Cao, Yifang Chen, Zhiqing Qin, Jiaming Bao, Shiya Wan, Hao Chen, Xiaoran Tang, Xiang Li, Xinyu Wang, Yuting Cheng, Yixia Li, Jie Zhang, Chang Liu, Xuguang Zhang, Yanan Wang, Yi Hu, Nanying Che, Xiaowei Xu, Hezhe Lu

**Affiliations:** 1 Department of Pathology, Beijing Chest Hospital, Capital Medical University, Beijing Tuberculosis and Thoracic Tumor Research Institute, Beijing, 101149 China; 2 Department of Thoracic Surgery, Beijing Tuberculosis and Thoracic Tumor Research Institute, Beijing Chest Hospital, Capital Medical University, Beijing, 101149 China; 3 State Key Laboratory of Organ Regeneration and Reconstruction, Institute of Zoology, Chinese Academy of Sciences, Beijing, 100101 China; 4 Beijing Institute for Stem Cell and Regenerative Medicine, Beijing, 100101 China; 5 Life Science and Medicine, University of Science and Technology of China, Hefei, 230026 China; 6 Department of Oncology, Shengjing Hospital of China Medical University, Shenyang, 117004 China; 7 Department of Thoracic surgery, Affiliated Hospital of Hebei University, Baoding, 071000 China; 8 Department of Oncology, Beijing Tuberculosis and Thoracic Tumor Research Institute, Beijing Chest Hospital, Capital Medical University, Beijing, 101149 China; 9 Innovation Centre of Ministry of Education for Development and Diseases, the Sixth Affiliated Hospital, School of Medicine, South China University of Technology, Guangzhou, 510006 China; 10 Key Laboratory of Cell Proliferation and Regulation Biology, Ministry of Education, College of Life Sciences, Beijing Normal University, Beijing, 100875 China; 11 Department of Intensive Care Unit, Beijing Chest Hospital, Capital Medical University, Beijing Tuberculosis and Thoracic Tumor Research Institute, Beijing, 101149 China; 12 National Key Laboratory for Novel Software Technology, Nanjing University, Nanjing, Jiangsu 210093 China; 13 Department of General Medicine Department, Eastern Medical District of Chinese PLA General Hospital, Beijing, 100094 China; 14 Department of Pathology, Affiliated Hospital of Hebei University, Baoding, 071000 China; 15 State Key Laboratory of Earth Surface Processes and Resource Ecology and Ministry of Education Key Laboratory for Biodiversity Science and Ecological Engineering, College of Life Sciences, Beijing Normal University, Beijing, 100875 China; 16 Department of Pathology and Laboratory Medicine, Perelman School of Medicine, University of Pennsylvania, Philadelphia, Pennsylvania 19104 USA

**Keywords:** Tumour biomarkers, Lung cancer

## Abstract

Neoadjuvant immunochemotherapy has shown promising results, with major pathologic response (MPR, ≤10% residual viable tumors [RVT]) as the primary outcome. However, %RVT showed limited predictive power in stratifying outcomes within the MPR and non-MPR groups. To identify a better prognostic marker, this study analyzed 200 non-small-cell lung cancer (NSCLC) samples after neoadjuvant PD-1 blockade combined with chemotherapy across three medical centers. Among these patients, 99 had necrotic regions in their residual lesions. We found that tumor-infiltrating lymphocytes in necrotic areas (nTILs) lose their cellular structure, but retained T-cell-specific antigens, making them detectable by immunohistochemistry. Regardless of PD-L1 status or lymph node metastasis, patients with high CD8^+^ nTIL density had significantly improved event-free survival (EFS) (hazard ratio [HR]: 0.08; 95% CI: [0.01–0.62]; *p* = 0.0019). Furthermore, CD8^+^ nTIL density improved prognostic predictions for patients within the MPR (*p* = 0.017) and non-MPR groups (*p* = 0.076). Radiological responses did not correlate with MPR, CD8^+^ nTIL density or EFS. 41.5% MPR cases were misclassified by radiological assessments. When compared with radiographic response and pathological response, CD8^+^ nTIL density outperformed these traditional parameters in approximating EFS. These findings demonstrate that the CD8^+^ nTIL density is a robust predictor of EFS in NSCLC patients treated with neoadjuvant immunochemotherapy and has great potential in guiding treatment decisions.

## Introduction

Non-small-cell lung cancer (NSCLC) remains a leading cause of cancer-related mortality worldwide.^1^ Although surgery is the standard treatment for early-stage disease, many patients experience relapse after surgery.^2,3^ Neoadjuvant systemic immunotherapy enables the effective activation of the immune system by leveraging the abundant release of tumor antigens, which promotes the immune surveillance and clearance of micrometastasis. Clinically, neoadjuvant immunochemotherapy has yielded promising results and become widely adopted for the treatment of NSCLC.^4–6^ Meanwhile, the gold standard for evaluating therapeutic benefit is improved overall survival;^7^ however, the collection of survival data takes as long as 5–10 years.^8^ To overcome this challenge, surrogate endpoints have been developed to allow for more efficient assessment of treatment efficacy, which can greatly reduce the time and cost of clinical trials and drug development. In light of this, an additional advantage of neoadjuvant therapy is that the examination of surgical specimens enables a pathological assessment of treatment efficacy within weeks. As a result, the evaluation of residual viable tumor (RVT) has been established as a surrogate endpoint in neoadjuvant treatment protocols.^2,8–11^ On the basis of the %RVT, patient outcomes can be classified as achieving complete pathologic response (pCR, 0% RVT), major pathologic response (MPR, less than or equal to 10% RVT) or non-major pathologic response (non-MPR, >10% RVT). However, %RVT fails to adequately stratify outcomes within the same pathologic response group.^12–15^ Importantly, approximately 20% of patients who achieve MPR or even pCR still experience recurrence within 3 years, highlighting the inadequacy of current metrics.^13,16^ This underscores the urgent need for new surrogate endpoints that reliably evaluate treatment efficacy, accurately predict patient outcomes, and guide treatment strategy decisions.

Geographic tumor necrosis is a distinct histopathological phenomenon characterized by confluent regions of dead tissue. Tumor necrosis is marked by the complete absence of viable tumor cells, the loss of normal tissue architecture and cellular structural details, and the accumulation of amorphous eosinophilic cellular debris. Historically, the presence of tumor necrosis is considered a hallmark of aggressive tumor biology. It is frequently associated with rapidly proliferating malignancies that outgrow their blood supply, leading to ischemic cell death, immunosuppression within the tumor microenvironment, and unfavorable clinical outcomes.^17,18^ Interestingly, neoadjuvant immunotherapy triggers immune-mediated tumor cell death, which may also result in necrosis. Although direct evidence linking immunotherapy to geographic necrosis remains limited, several clinical observations support this association. For instance, studies in patients with melanoma and hepatocellular carcinoma have reported a marked increase in necrotic tumor areas following neoadjuvant immune checkpoint blockade;^19,20^ this may reflect successful immune cell-mediated killing of tumor, providing valuable insights into treatment efficacy and tumor–immune interactions.

Tumor-infiltrating lymphocytes (TILs) are integral mediators of antitumor immunity and have been established as key determinants of response to immunotherapy across multiple cancer types.^20–23^ Studies have shown that the density and spatial distribution of TILs in pre-treatment biopsies can be used to predict the efficacy of immunotherapy. However, following neoadjuvant treatment, the tumor microenvironment often becomes extensively infiltrated by lymphocytes, thereby compromising the discriminative power of TIL density as a prognostic biomarker.^19,24^ After eradicating tumor cells, T lymphocytes undergo caspase-dependent or caspase-independent cell death.^25^ Interestingly, emerging evidence suggests that although lymphoid cells within regions of tumor necrosis lose their structural integrity and become undetectable by conventional hematoxylin and eosin (H&E) staining, certain T-cell specific antigens may still persist. This preservation of antigenic markers enables the identification of TILs within necrotic areas (nTILs) via immunohistochemistry (IHC), offering a potential window to study the immune activities that occurred within necrotic tumors.^26,27^ These nTILs, having mediated tumor cell killing, represent one of the most direct indicators of host response to immunotherapy. Studies in melanoma have demonstrated that the presence of CD3^+^ and CD8^+^ nTILs is associated with pathologic response following neoadjuvant anti-PD-1 therapy.^19^ However, due to limited sample sizes, previous studies were unable to comprehensively evaluate the correlation between nTILs and other established prognostic markers in tumors. The clinical relevance and predictive value of nTILs in NSCLC remain unexplored.

Here, our analysis of CD3 and CD8 IHC staining in post-treatment samples from 200 NSCLC patients reveals a highly heterogeneous spatial distribution of TILs in the residual tumor bed, and provides the first comprehensive evaluation of TILs in tumor, regression and necrotic areas. Our findings indicate that, while CD3^+^ and CD8^+^ TIL density within the tumor and regression areas exhibited little correlation with event-free survival (EFS), CD8^+^ TIL density in necrotic areas demonstrated a strong association with patient prognosis. Furthermore, CD8^+^ nTIL density served as a robust predictive marker for patient outcomes, regardless of baseline tumor PD-L1 expression status, the presence of lymph node metastasis prior to surgery, or the achievement of MPR. Our findings demonstrated that CD8^+^ nTIL density predicted EFS with greater precision than current metrics, such as pathological and radiological response. CD8^+^ nTIL has the potential to serve as an independent predictor or be used in combination with pathological response to guide treatment decisions.

## Results

### Patient population

**Fig. 1:**
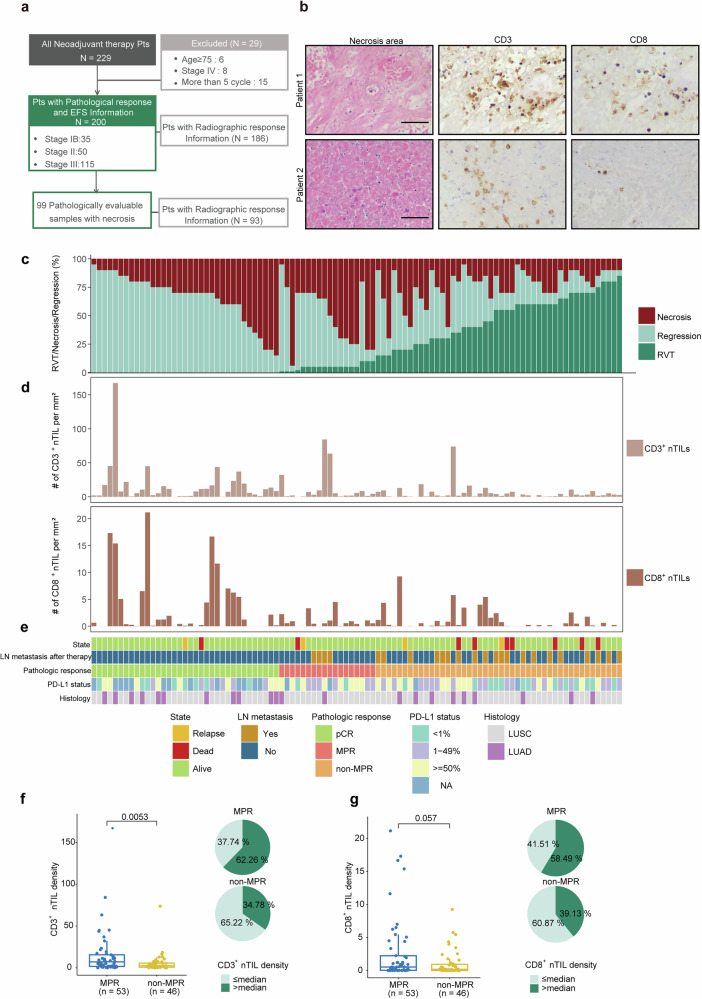
Tumor pathologic features and nTIL density. **a** Clinical profile of patients included in the study. Database lock: December 12, 2024; minimum follow-up: 12 months; median follow-up: 25 months. **b** Representative images showing the immunohistochemical staining of CD3 and CD8 in regions of tumor necrosis in samples from two NSCLC patients. Scale bars, 200 μm. **c** Pathologic features (percentage of RVT, regression and necrosis) in patients with tumor necrosis (*n* = 99). **d** CD3^+^ nTIL and CD8^+^ nTIL density in patients listed in (**c**). **e** Tumor PD-L1 status, LN metastasis, pathological response and outcome in patients listed in A. “LN metastasis after therapy” was confirmed through pathological diagnosis of the surgical samples following neoadjuvant therapy. **f** and **g**. CD3^+^ (**f**) and CD8^+^ (**g**) nTIL density for each patient in the MPR (*n* = 53) and non-MPR (*n* = 46) groups (left), and pie charts show the proportion of patients with high or low nTIL density in the MPR and non-MPR groups (right)

A total of 229 NSCLC patients who underwent surgery following neoadjuvant PD-1 blockade plus chemotherapy across 3 medical centers were eligible for inclusion; of these, 29 patients were excluded from the analysis due to distant metastasis, age at the time of diagnosis, or excessive treatment cycles. Among the 200 patients whose clinicopathological data were available, 99 with necrotic areas were eligible for nTIL evaluation, and 186 with radiological response data were eligible for comparative analysis. All eligible patients were included in the relevant analyses without any other selection criteria. The database was locked on December 12, 2024; the minimal follow-up was 12 months, and the median follow-up (survivors) was 26 months (Fig. 1a and Supplementary Fig. 1a). The cohort consisted of 160 (80%) patients diagnosed with squamous cell lung carcinoma (LUSC) and 40 (20%) patients with lung adenocarcinoma (LUAD). The baseline characteristics of the pathologically evaluable patient population are summarized in Table 1.

**Table 1:** Clinical cohort description

**Sex**	
Male	177 (88.5%)
Female	23 (11.5%)
**Age (median** = **63)**	
>65	75 (37.5%)
≤65	125 (62.5%)
**Tumor type**	
Adenocarcinoma	40 (20%)
Squamous cell carcinoma	160 (80%)
**Histological response**	
pCR	81 (40.5%)
MPR	37 (18.5%)
non-MPR	82 (41%)
**Smoking status**	
ever	158 (79%)
never	42 (21%)
**PD-L1**	
<1%	42 (21.0%)
1–49%	63 (31.5%)
≥50%	54 (27.0%)
Missing	41 (20.5%)
**LN Metastasis (after therapy)**	
Yes	112 (56%)
No	88 (44%)
**Stage**	
IB	35 (17.5)
II	50 (25%)
III	115 (57.5%)
**Treatment cycle**	
1	6 (3%)
2	122 (61%)
3	51 (25.5%)
4	18 (9%)
5	3 (1.5%)

### Pathological response and TILs

Pathological assessment was performed according to the pan-tumor immune-related pathologic response criteria (irPRC).^28^ Specifically, features including %RVT, tumor regression and necrosis were evaluated in the tumor bed (i.e., the area where the tumor was previously located) (Supplementary Fig. 1b). Pathology revealed that 59% of patients achieved pCR or MPR, while 41% of patients had non-MPR (Supplementary Fig. 2a and Supplementary Table 1).

To determine the relationship between TILs and pathological response, we performed CD3 and CD8 IHC staining on tumor samples from our NSCLC cohort. Consistent with previous studies^29–31^, in patients with MPR, the number of TILs was relatively high in both the tumor and regression regions. However, the correlation was not statistically significant (Supplementary Fig. 2b, c). Interestingly, although remnants of T lymphocytes within necrotic areas no longer retained their cellular structure, traces of common T-cell-specific antigens still remained, allowing the detection of nTILs. The density of nTILs varied across different NSCLC patients (Fig. 1b). To determine the correlation between patient outcome and distinct subsets of nTILs, we counted the number of CD3^+^ nTILs and CD8^+^ nTILs per unit area of necrosis. Patients with a higher %RVT had significantly lower nTIL density (Fig. 1c, d and Supplementary Fig. 3a), and patients with unfavorable clinical outcomes (recurrence or death) had a significantly lower CD8^+^ nTIL density (Fig. 1e). In addition, we found that patients with elevated levels of CD8^+^ nTILs and CD3^+^ nTILs were more prevalent in the MPR group than in the non-MPR group (Fig. 1f, g). Moreover, little correlation was observed between CD8^+^ nTIL density and PD-L1 status or lymph node (LN) metastasis (Supplementary Table 2).

LUSC was the most common cancer subtype in our cohort (*n* = 160). Among the remaining 40 LUAD samples, 22 contained necrotic areas, where the mean CD8^+^ nTIL density was significantly greater than that in LUSC. Notably, only one patient in this group experienced tumor recurrence, and no CD8^+^ nTILs were detected in his tumor tissue (Supplementary Fig. 3b–g and Supplementary Table 2).

### Pathologic response and EFS

**Fig. 2:**
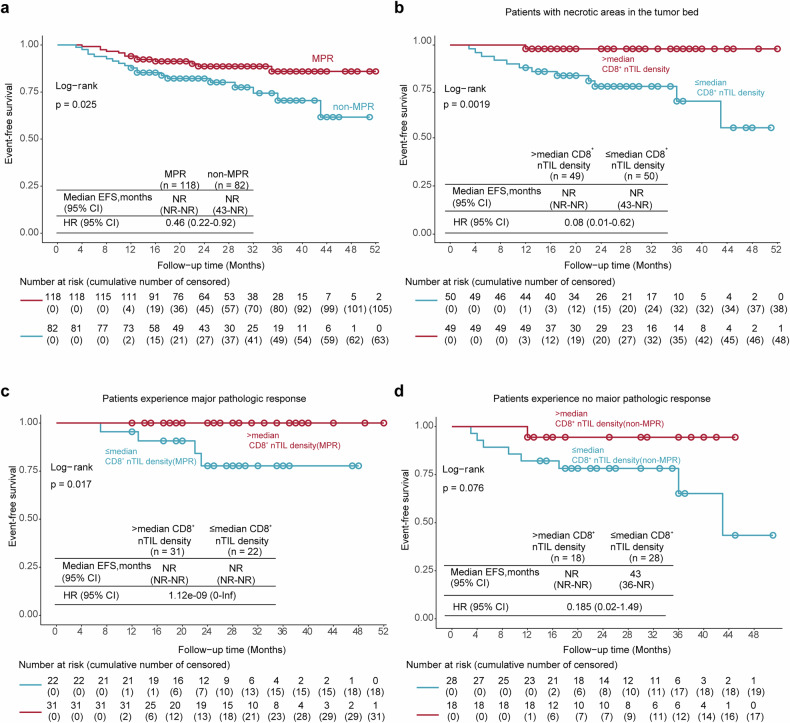
Association between CD8^+^ nTILs and EFS. **a** Kaplan–Meier curves for EFS in the MPR group vs non-MPR group. A major pathological response was defined as having a <10% RVT. Patient number: 200; Event number: 32; median follow-up: 26 months (survivors); HR hazard ratio. **b** Kaplan–Meier curves for EFS grouped by CD8^+^ nTIL density. The cutoff was set by the median number of lymphocytes per 1 mm^2^ (0.41 CD8^+^ nTIL counts per mm^2^). Patient number: 99; Event number: 13; median follow-up (survivors): 26.5 months. **c**, **d** Kaplan‒Meier curves for EFS grouped by CD8^+^ nTIL density in the MPR group (**c**) and non-MPR group (**d**). MPR group number: 53; Event number: 4; median follow-up: 27 months; non-MPR group number: 46; Event number: 9; median follow-up (survivors): 26 months

Previous studies have shown that an elevated %RVT was associated with poor prognosis in NSCLC patients receiving neoadjuvant immunotherapy.^29,32,33^ Patients with MPR demonstrated significantly improved EFS compared with those with non-MPR (Fig. 2a; EFS rates for MPR versus non-MPR: 85.9% versus 61.7%, respectively). *HR* = 0.46, 95% CI: [0.22–0.92], *p* = 0.025. The associations between other pathological parameters and EFS are presented in Supplemental Table 3. However, %RVT had limited predictive value, as it failed to stratify relapse risk further within the MPR and non-MPR groups (Supplementary Fig. 4a). Unlike what was observed in previous studies,^29,32,33^ %regression did not significantly correlate with EFS (Supplementary Fig. 4b). Patients with >30% necrosis after neoadjuvant therapy had improved prognoses, although the differences were not statistically significant (Supplementary Fig. 4c). Remarkably, the 4 year EFS rate for patients with >30% necrosis was 96.8%, compared to 64% for those with <30% necrosis. To further delineate this correlation, %necrosis in patients with MPR and non-MPR were compared (Supplementary Fig. 4d). The median %necrosis in MPR patients was 30%, which was significantly greater than %necrosis in non-MPR patients, suggesting that in NSCLC, neoadjuvant immunochemotherapy may lead to therapeutic tumor necrosis.

### CD8^+^ and CD3^+^ nTILs and EFS

When assessing the relationship between TILs and EFS, we initially reported that patients with moderate or brisk CD3^+^/CD8^+^ TILs in the tumor and stromal regions presented a lower risk of recurrence compared to those with absent/minimal TILs. However, the difference was not statistically significant, and the ability to accurately predict recurrence risk on the basis of TILs remains limited (Supplementary Fig. 5).

Importantly, TIL density within necrotic areas was strongly correlated with patient prognosis. Patients with high CD8^+^ nTIL density, where the cutoff was the median density of CD8^+^ nTILs (median = 0.41 nTIL/mm^2^), had significantly longer EFS (HR = 0.08, *p* = 0.0019; Fig. 2b). While patients with higher CD3^+^ nTIL density also had longer EFS, the correlation was not statistically significant (Supplementary Fig. 7a). Using a univariate Cox proportional hazard model for EFS that incorporated CD8^+^ nTILs and pathologic response as factors, we found that high CD8^+^ nTIL density was associated with better outcomes in both the MPR (HR = 1.12e-09, 95% CI: [0-Inf], *p* = 0.017) and non-MPR (HR = 0.185, 95% CI: [0.02–1.49], *p* = 0.076) groups (Fig. 2c, d). These findings suggest that CD8^+^ nTILs serve as a robust indicator of EFS, complementing the pathological response in predicting neoadjuvant therapy outcome.

### LN involvement

**Fig. 3:**
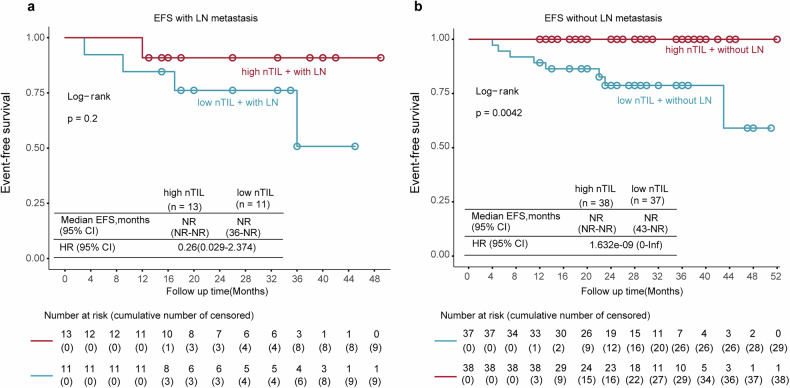
Treatment efficacy in patients with or without LN involvement. **a**, **b** Kaplan‒Meier curves showing EFS by CD8^+^ nTILs in patients with (**a**) or without (**b**) LN involvement. LN metastasis group number: 24; event number: 5; median follow-up: 33 months; no LN metastasis group number: 75; event number: 8; median follow-up (survivors): 26 months. HRs were not computed because of the low number of patients in the LN metastasis subgroups

To determine whether the presence or absence of residual tumor in the lymph node (LN) affects outcome prediction by CD8^+^ nTIL density following neoadjuvant therapy, we first compared the EFS between patients grouped by their status of LN metastasis and observed a modest improvement in EFS in the group of patients without residual tumor in the LN (Supplementary Fig. 6d). When we focused on the subpopulation of patients with necrosis and evaluated the correlation between CD8^+^ nTILs and EFS between groups with different LN metastatic status, we found that the association between high CD8^+^ nTILs and longer EFS was consistent in both arms regardless of LN involvement (Fig. 3a, b). Notably, no patients with high CD8^+^ nTILs and no LN residual tumor experienced tumor recurrence at the time of database lock.

### CD8^+^ nTILs versus pathological and radiological responses

**Fig. 4:**
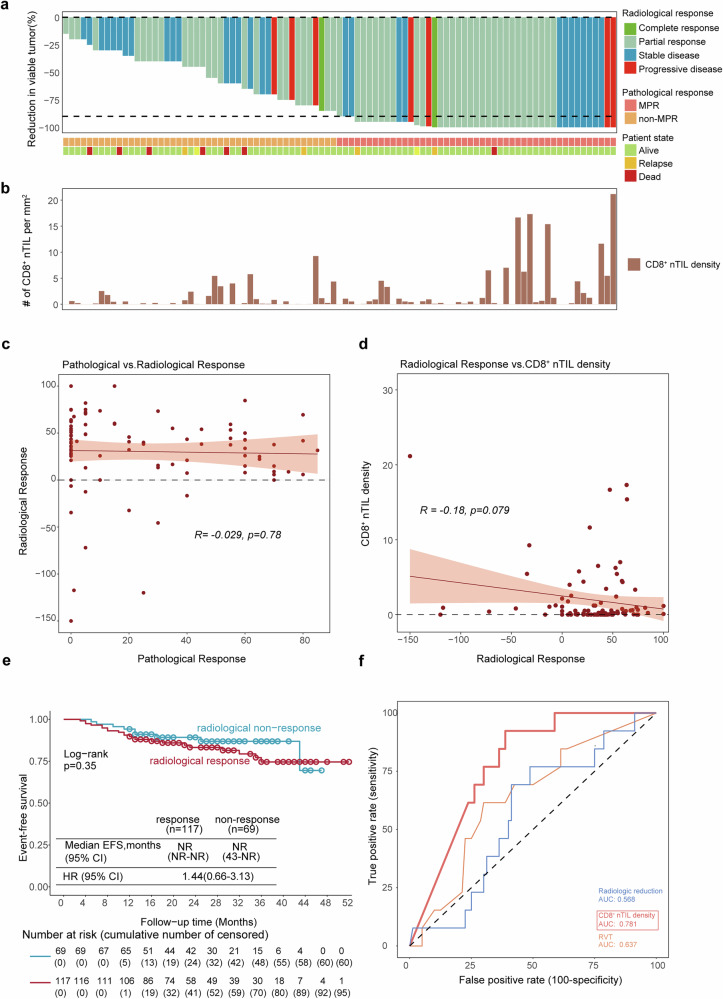
Relationships between the nTILs and pathological and radiological responses. **a** Waterfall chart illustrating the % reduction in viable tumors in patients with necrosis, as assessed by pathological examination of surgically resected tumor samples. The radiological response of each patient was also evaluated and denoted here with the different colors of the bars (CR-complete radiological response; PR-partial radiological response; SD-stable disease; PD-progressive disease). The dashed line represents the cutoff for major pathological response (90% tumor regression); the pathologic response and patient outcome are color-coded and listed beneath the chart. **b** CD8^+^ nTIL density in patients with necrosis. **c**, **d** The correlation between the reduction in tumor size as determined by radiological assessment (according to RECIST criteria, version 1.1) and the pathologic response (**c**) or CD8^+^ nTIL density (**d**) in each surgical specimen. RECIST Response Evaluation Criteria in Solid Tumors, version 1.1. **e** Kaplan‒Meier curves showing EFS by radiological response. Patient number: 186; Event number: 31; median follow-up (survivors): 26 months; (**f**) Prediction of patient outcome on the basis of different parameters. The value of the AUC represents the prediction quality

Pathological and radiological responses are commonly used as surrogates for evaluating the treatment efficacy of neoadjuvant therapy. In our NSCLC cohort, a comparison of radiological and pathological assessments of residual disease after neoadjuvant therapy revealed notable discrepancies, as radiological imaging often fails to predict treatment outcomes. For example, in a representative case (Supplementary Fig. 7a), radiological assessment revealed only a slight reduction in tumor size, while the resected tumor was pathologically classified as pCR, accompanied by a high density of CD8^+^ nTILs. Although patients with better radiological responses typically exhibit a lower %RVT, the correlation between pathological and radiological responses was minimal (*R* = −0.029; *p* = 0.78). A total of 45 out of the 118 patients who achieved MPR or pCR did not exhibit a radiological response according to RECIST 1.1 (Fig. 4a, c and Supplementary Fig. 7b). Similarly, a weak correlation was observed between CD8^+^ nTIL density and radiological response (*R* = −0.18, *p* = 0.079; Fig. 4b, d and Supplementary Fig. 7c). In addition, the radiological response was not correlated with EFS, with no significant differences in EFS between responders and non-responders (Fig. 4e).

By evaluating the effects of CD8^+^ nTILs, CD3^+^ nTILs, pathological response, and radiological response on survival via a univariate model in the path-evaluable cohort^13^, we found that CD8^+^ nTIL density was the only factor significantly associated with outcomes in patients with necrosis (Table 2). CD8^+^ nTILs demonstrated superior predictive power, as confirmed by a high AUC value in the receiver operating characteristic curve (ROC) analysis (Fig. 4f). These findings highlight CD8^+^ nTIL density as a robust and reliable predictor of patient outcomes, outperforming traditional radiological and pathological metrics.

**Table 2:** Association of EFS and survival surrogates for patients undergoing treatment with neoadjuvant PD-1 blockade plus chemotherapy

Path-evaluable population				
Survival surrogate	No.	HR (EFS)	95% CI	*p*-value
Radiographic response by RECIST 1.1 : responders versus non-responders	57 (responders) 36 (non-responders)	2.4	0.66–8.8	0.183
Pathological response: pCR versus non-pCR	35 (pCR) 64 (non-pCR)	0.35	0.078–1.6	0.175
Pathological response: MPR versus non-MPR	53 (MPR) 46 (non-MPR)	0.36	0.11–1.8	0.092
CD8^+^ nTIL density: High CD8^+^ nTIL density versus Low CD8^+^ nTIL density	49 (High) 50 (Low)	0.081	0.01–0.62	0.016*
CD3^+^ nTIL density: High CD3^+^ nTIL density versus Low CD3^+^ nTIL density	49 (High) 50 (Low)	0.31	0.086–1.1	0.077

*p-value < 0.05

## Discussion

In this study, we found that high levels of CD8^+^ nTILs were strongly associated with improved EFS in NSCLC patients treated with neoadjuvant immunochemotherapy. The predictive value of CD8^+^ nTILs was independent of clinical indicators such as PD-L1 status, lymph node metastasis, and tumor grade, and elevated CD8^+^ nTIL levels predicted improved EFS regardless of whether the MPR was achieved. Furthermore, the CD8^+^ nTIL density outperformed radiological and pathological responses in predicting treatment outcomes. These findings highlight the critical role of the CD8^+^ nTILs in immunochemotherapy response and underscore their potential as a reliable predictor in neoadjuvant immunochemotherapy.

Currently, the prediction of treatment efficacy and patient prognosis can be carried out using benchmarks within weeks after receiving neoadjuvant therapy,^8^ which greatly accelerates drug development and facilitates regulatory approval. Pathological response commonly acts as a surrogate endpoint for patient outcomes in multiple cancer types.^9,10,34^ This endpoint has been successfully utilized to obtain regulatory approvals from the FDA and is currently one of the most cost-effective methods for predicting patient prognosis following neoadjuvant therapy.^35^ However, while the pathological response is correlated with EFS, the prediction is not entirely reliable. For example, 14.1% of NSCLC patients with pCR or MPR experience recurrence within 4 years, whereas 61.7% of patients with non-MPR are recurrence-free within the same timeframe. Furthermore, the %RVT struggled to stratify patient outcomes within the same pathological subgroup (Supplementary Fig. 4a). TILs in pre-treatment samples have been implicated in predicting response to neoadjuvant immunochemotherapy. However, it remains unclear whether the density and spatial distribution of TILs in post-treatment surgical specimens can predict the risk of tumor recurrence. TIL populations are highly heterogeneous, and different lymphocyte subsets within the tumor microenvironment often play opposing roles in tumor immunity. Since conventional H&E staining cannot distinguish between these functionally distinct TIL subpopulations, we used CD3 and CD8 IHC staining to quantify and spatially resolve cytotoxic T cells. Our analysis revealed considerable spatial heterogeneity in CD3^+^ and CD8^+^ TIL distribution, with generally higher density observed in regression compared to tumor regions. Patients with moderate or brisk CD3^+^/CD8^+^ TILs in the tumor or regression regions presented a lower risk of recurrence compared to those with absent/minimal TILs. However, these correlations were not statistically significant, which might be due to the relatively high TIL density in the residual lesions of most patients who received neoadjuvant immunochemotherapy. Our analysis suggested that CD8^+^ nTIL density after neoadjuvant immunotherapy may improve the prediction of patient outcomes in patients whose pathological response alone is insufficient. Among patients who achieved MPR, those with low CD8^+^ nTIL densities still experienced a considerable risk of recurrence. Conversely, in patients who did not achieve MPR, high CD8^+^ nTIL densities nonetheless indicated favorable prognoses.

The use of tumor necrosis as a predictor of neoadjuvant treatment efficacy remains controversial.^10,13,36^ In our analysis, although a tentative association is observed between the %necrosis and certain prognostic endpoints, the correlation doesn’t reach statistical significance. These results suggest that necrosis alone is an inadequate and unreliable predictor of treatment outcomes. One major complicating factor is the inherent presence of spontaneous necrosis in untreated NSCLC, which becomes more prevalent in advanced-stage disease. It is crucial challenging to differentiate between functionally disparate forms of necrosis by pathological test. Spontaneous necrosis contributes to tumor progression, but treatment-induced necrosis reflects therapeutic efficacy. Immunotherapy relies on TILs to achieve effective tumor cell eradication; therefore, we hypothesize that necrosis induced by immunotherapy is immunologically active and characterized by a significant enrichment of nTILs. This feature may serve to distinguish it from spontaneous necrosis, which typically results from ischemic stress without substantial CD8^+^ T-cell infiltration. This hypothesis is supported by our findings of a strong positive correlation between the density of CD8^+^ nTILs and the degree of pathological response.

We observe a limited correlation between radiological response and establish clinical endpoints, including pathological response and EFS. This finding underscores the potential inadequacy of relying solely on conventional imaging-based assessments as surrogates for treatment efficacy, which may lead to a skewed or incomplete evaluation of patient outcomes. This discrepancy may arise from the mechanism of action of immunotherapy, which elicits distinct response patterns mediated by T cells within the tumor microenvironment. These phenomena often complicate the radiological interpretation of tumor size and density after treatment, limiting the accuracy of image-based metrics in capturing true biological and clinical benefits.

We acknowledge several limitations in our study. First, variations in nTIL counts from different medical centers may impact correlation assessments for patients. To address this, we plan to develop machine learning-based software for nTIL density measurement, which will standardize the evaluation process and enable integrated analyses across independent studies from different research groups. Second, we select a median value of 0.41 (CD8^+^ nTILs per mm^2^) as the cutoff, which result in a robust predictive model without further experimenting with other cutoff values. Third, some data are missing for a small group of patients, including PD-L1 status for 41 patients and radiographic response information for 14 patients; this may have reduced the statistical power of the analysis. In addition, the cohort of 200 patients remains not enough, limiting our ability to systematically integrate all relevant pathological features. Consequently, it is unable to determine the precise contribution of each prognostic factor to risk prediction or to develop a robust and optimized predictive model. In the future, a larger cohort study will enable us to determine the optimal cutoff value and further validate the associations between CD8^+^ nTILs and other clinical markers.

In conclusion, our study establishes the first global benchmark for characterizing nTILs in response to neoadjuvant PD-1 blockade combined with chemotherapy in NSCLC patients. We demonstrate that CD8+ nTIL density, as quantified by standardized IHC staining, represents a clinically feasible, reproducible, and easily implementable method for predicting treatment outcomes. Its practicality and reliability support its potential applicability not only in NSCLC but also as a generalizable biomarker across other solid tumor types. Thus, CD8^+^ nTIL density hold promise as an independent predictor or in combination with pathological response to guide neoadjuvant immunochemotherapy in perisurgical clinical trials.

## Materials and methods

### Patients and treatment

This study is a retrospective analysis based on patient data from clinical trials and standardized treatments from three medical centers. Patient outcomes were documented through routine post-operative follow-up. The study was conducted in accordance with the ethical guidelines and research protocol of Beijing Chest Hospital (Approval Number: LW-2024-022), Shengjing Hospital of China Medical University (2024PS1588K), and the Affiliated Hospital of Hebei University (HDFYLL-KY-2024-188). Ethical approval for the study was obtained following a comprehensive review by the respective ethics committees. Clinical and histopathological data from three centers (229 patients) were gathered. Among them, 8 patients (stage IV) with distant metastasis, 6 patients over the age of 75 at the time of diagnosis, and 15 patients who received >5 treatment cycles were excluded. All 200 patients meeting the inclusion criteria, with pathological diagnoses and EFS data, were enrolled in the study. Among them, 99 with necrotic areas were suitable for nTIL analysis, and 186 with radiological response data were available for comparative analysis. Patients received anti-PD-1 antibody at a dose of 360 mg combined with platinum or paclitaxel chemotherapy every 3 weeks for one to five cycles before undergoing definitive surgery within 4–6 weeks after completing neoadjuvant treatment. Patient characteristics, such as PD-L1 status (PD-L1 information was missing or undetectable for 41 patients), disease stage, and sex at baseline and after treatment, were collected.

### Response assessment

Pathologic response was defined according to blinded independent pathologic review. Following the relevant guidelines from the collaborating hospitals, two pathologists reviewed each patient’s pathological diagnosis independently. If there was a discrepancy, a third pathologist combined the first two to provide a final diagnosis. For all pathologically evaluable samples from patients who underwent definitive surgery after neoadjuvant treatment, the percentages of RVT, regression and necrosis were quantified using a pan-tumor scoring system^13^. To be exact, pCR was defined as the complete absence of the RVT cross-sectional tumor bed. MPR was characterized by ≤10% RVT, whereas non-MPR was defined as >10% RVT. Radiological response was assessed based on RECIST version 1.1 using CT or MRI performed before and after treatment. Patients were classified as having an overall radiological response if the imaging results indicated a complete response (CR) or partial response (PR). Conversely, those with stable disease (SD) or progressive disease (PD) were categorized as having no overall radiological response^13^.

EFS was assessed by blinded independent central review. EFS was defined as the time from the randomization to occurrence of one of the following events: progressive disease after surgery, distant metastasis or death from any cause, in per blinded independent central review.

### CD3 and CD8 immunohistochemistry

Formalin-fixed, paraffin-embedded (FFPE) tissue blocks were deparaffinized and rehydrated by sequentially passing them through changes in xylene and graded ethanol solutions, and 5 μm FFPE slides underwent heat-induced epitope retrieval via BOND Epitope Retrieval solution (Leica Microsystems AR9961 or AR9940). To block endogenous peroxidase activity, the slides were incubated in 3% hydrogen peroxide solution before being treated with the primary antibody (CD3, 1:200, Dako M0701, clone 2B11^+^PD7/26; CD8, 1:200, Dako M0701, clone 2B11^+^PD7/26). Staining was carried out via the Bond Polymer Refine Detection System (Leica Microsystems DS9800), and the stained slides were counterstained with hematoxylin before review. For each patient, 10 to 50 paraffin blocks were prepared from the surgical samples. Based on preliminary pathological diagnosis, the slice representing the most typical necrotic tumor area was selected.

The necrotic regions and the total number of nTILs within that slice were then counted separately by two pathologists. The average of the two independent measurements was then calculated and divided by the area of necrosis to obtain the nTIL count per unit area. The median CD3^+^ nTIL density was 4.23 cells/mm², and the CD8^+^ nTIL density was 0.41 cells/mm². In 49 patients with a CD3^+^ nTIL density below 4 cells/mm², the mean was 1.38, with a standard error of 0.155. For the 50 patients with a density exceeding 4 cells/mm², the mean was 20.1, with a standard error of 3.94. In 76 patients with a CD8^+^ nTIL density <2 cells/mm², the mean was 0.34, with a standard error of 0.05. For the 23 patients with a density exceeding 2 cells/mm², the mean was 7.22, with a median of 5.43 and a standard error of 1.13.

### Statistical analysis

Following Deutsch et al.^13^ patient and disease characteristics were summarized as categorical or continuous variables using standard descriptive statistics. To uncover potential relationships, the frequencies (or proportions) were computed for categorical variables and compared across groups, and the significance of comparisons was evaluated through the chi-square test (chi-square test). Differences in continuous variables across groups were visualized via box plots, with *p*-values calculated via the Wilcoxon rank-sum test (Wilcox test).

The statistical method for EFS was based on the work of previous studies.^13,20^ EFS after neoadjuvant therapy was analyzed via the Kaplan‒Meier method, and the log-rank test was used to compare survival curves between two or more groups. If a median survival time was reached, it was displayed in the graph; otherwise, it was denoted as NR, and the 95% confidence interval for the median survival time was calculated when available. HR was calculated via a univariate or multivariate Cox proportional hazards regression model. The two-sided 95% confidence interval (95% CI) was calculated via the ‘coxph’ function of the R package ‘survival’ with default parameters. When multiple survival curves were compared, the ‘pairwise_survdiff’ function was used to obtain pairwise *p*-values.

To explore the correlation between radiological response and other diagnoses, a scatter plot was drawn together with a fitted linear regression line, and the Pearson correlation coefficient was also calculated via the ‘cor’ function.

Receiver operating characteristic (ROC) curve^13^ analysis was conducted to evaluate the predictive ability of different diagnostic metrics. The area under the ROC curve (AUC)^13^ metric, a key measure of prediction performance, was computed via the R package multi. All the statistical analyses were conducted via R (version 4.3.1).

## Supplementary information

Sigtrans_Supplementary_Materials

Supplemental Figure Legends

## Data Availability

The data used in this study are deposited on the China National Center for Bioinformation under study ID OMIX010789. Deidentified and anonymized data will be made available through a secure portal to qualified researchers upon reasonable request to the corresponding authors. Proposals will be reviewed to demonstrate researchers’ relevant experience and address any potential conflicts of interest. A material transfer agreement is used to facilitate the transfer of any data that can be shared.
