# Supplementary material for: CD8+ TILs in necrotic tumors after neoadjuvant immunochemotherapy predict outcomes in non-small-cell lung cancer patients
Source: Signal Transduct Target Ther. 2025 Oct 9;10:335. doi: 10.1038/s41392-025-02435-0 (PMC12508027; doi:10.1038/s41392-025-02435-0)
Supplement: Supplementary file 2 — Supplemental Figure Legends [file 41392_2025_2435_MOESM2_ESM.docx]

**Supplemental Figure Legends：**

**Supplemental Figure 1. Sample collection and illustrative representation of irPRC scoring.**

**a.** Timeline for radiographic imaging and the collection of tumor tissues before and after neoadjuvant therapy.

**b.** This representative image is an example of what is observed in the pathologic evaluation of a tumor sample. In the resected tumor specimen, geographic tumor necrosis is marked by the absence of living cells, the disappearance of cellular structures, and the presence of shapeless, cellular debris.

**Supplemental Figure 2. Tumor pathologic features and TIL density in RVT and regression areas.**

**a.** Pathologic features and outcome for all NSCLC patients. “LN metastasis after therapy” was confirmed through pathological diagnosis of the surgical specimens following neoadjuvant therapy.

**b.** The relationship between CD3^+^ and CD8^+^ TIL density in regression areas and pathologic response. Pie charts show the proportion of patients with absent/minimal, moderate, or brisk of TILs in the MPR and non-MPR groups.

**c.** The relationship between CD3^+^ and CD8^+^ TIL density in RVT and pathologic response. Pie charts show the proportion of patients with absent/minimal, moderate, or brisk of TILs in the MPR and non-MPR groups.

**Supplemental Figure 3. Tumor pathologic features and nTIL density in LUSC and LUAD.**

**a.** CD3^+^ nTIL and CD8^+^ nTIL density by %RVT categories. Patients were grouped by %RVT (n=118 for 0-10% RVT, n=41 for 11-50% RVT, n=41 for >50%RVT).

**b.** Pathologic features (top) and outcome (bottom) for LUAD patients without necrosis (n=18).

**c.** Pathologic features (top), outcome (middle), and CD3^+^/CD8^+^ nTIL density (bottom) for LUAD patients with necrosis (n=22).

**d.** Pathologic features (top) and outcome (bottom) for LUSC patients without necrosis (n=83).

**e.** Pathologic features (top), outcome (middle), and CD3^+^/CD8^+^ nTIL density (bottom) for LUSC patients with necrosis (n=77).

**f.** CD8^+^ nTIL density for each patient in LUSC (n=77) and LUAD (n=22) groups.

**g.** Kaplan-Meier curves comparing EFS between LUAD (n=22) and LUSC (n=77). Patients with necrosis after treatment are listed. Patients number: 99; Event number: 13; median follow-up (survivors): 26.5 months

**Supplemental Figure 4. Regression, necrosis and EFS**

**a.** Kaplan–Meier curves showing EFS by %RVT categories. Patients were grouped by %RVT, where the cut-offs are set to be >50%RVT, 11-50%RVT, or 0-10%RVT. Patients number: 200; Event number: 32; median follow-up (survivors): 26 months.

**b.** Kaplan–Meier curves for EFS according to %regression.

**c.** Kaplan–Meier curves for EFS according to %necrosis. Patients were divided into two groups first: with and without necrosis. In the necrosis group, a median necrosis percentage (30%) was used as the cutoff.

**d.** % necrosis for each patient in MPR (n=53) and non-MPR (n=46) groups (left), and Pie charts show the proportion of patients with high (>30%) or low (<30%) %necrosis in the MPR and non-MPR groups (right).

**Supplemental Figure 5. Association between TIL in RVT, regression areas and EFS.**

**a.** and **b.** Kaplan-Meier curves for EFS grouped by CD3^+^ (**a**) and CD8^+^ (**b**) TIL density in RVT areas. patients were grouped by TILs density: absent/minimal, moderate, or brisk. Patients number: 119; Event number: 24; median follow-up (survivors): 27 months.

**c.** and **d.** Kaplan-Meier curves for EFS grouped by CD3^+^ (**c**) and CD8^+^ (**d**) TIL density in regression areas. Patients number: 200; Event number: 32; median follow-up (survivors): 26 months.

**Supplemental Figure 6. Correlation between CD3^+^ nTIL density and EFS.**

**a.** Kaplan-Meier curves for EFS according to CD3^+^ nTIL density. Patients were grouped by CD3^+^ nTIL density, where the cut-off is set to be the median lymphocytes number per 1 mm^2^ (4.23 CD3^+^ nTILs counts per mm^2^). Patients number: 99; Event number: 13; median follow-up (survivors): 26.5 months;

**b.** and **c.** Kaplan-Meier curves for EFS according to CD3^+^ nTIL density in MPR group (**b**) and non-MPR group (**c**). MPR group number: 53; Event number: 4; median follow-up: 27 months; non-MPR group number: 46; Event number: 9; median follow-up (survivors): 26 months.

**d.** Kaplan-Meier curves showing EFS by LN involvement. Among the 154 patients, no lymph node metastasis was found, while tumor residue was detected in the lymph nodes of 46 patients. LN involvement refers to pathological evidence of lymph node disease at the time of resection following neoadjuvant therapy. Patients number: 200; Event number: 32; median follow-up (survivors): 26 months.

**Supplemental Figure 7. Relationship between nTILs and other clinical biomarkers.**

**a.** A representative case illustrates the potential discrepancy between radiological response, pathologic response and nTIL density. On the left, the pre-treatment (top) and post-treatment (bottom) CT scans reveal little radiological response, showing only a little reduction in tumor size. Conversely, pathologic assessment in the middle shows this patient achieved pCR. The IHC staining image displays a significant abundance of CD8^+^ and CD3^+^ nTILs within the necrotic regions.

**b.** Waterfall chart illustrating the % reduction in viable tumor in patients without necrosis, assessed by pathological examination of surgically resected tumor samples. The radiological response of each patient was also evaluated and denoted here with the different coloring of the bars (CR-complete radiological response; PR-partial radiological response; SD-stable disease; PD-progressive disease). The dashed line represents the cutoff for major pathological response (90% tumour regression); the pathologic response and the patient outcome are color-coded and listed beneath the chart.

**c.** The correlation between the reduction in tumor size as determined by radiological assessment (according to RECIST criteria, version 1.1) and CD3^+^ nTIL density in each surgical specimen. RECIST=Response Evaluation Criteria in Solid Tumours, version 1.1.
